# Advances in lung bioengineering: Where we are, where we need to go, and how to get there

**DOI:** 10.3389/frtra.2023.1147595

**Published:** 2023-04-17

**Authors:** Tiffany Hsiung, Les James, Stephanie H. Chang, Travis C. Geraci, Luis F. Angel, Justin C. Y. Chan

**Affiliations:** ^1^ Department of Cardiothoracic Surgery, NYU Langone Health, New York, NY, United States; ^2^ Department of Cardiothoracic Surgery, NYU Transplant Institute, NYU Langone Health, New York, NY, United States

**Keywords:** lung transplant, tissue engineering, ex vivo lung perfusion, bioreactor, tissue scaffolds, extracorporeal membrane oxygenation

## Abstract

Lung transplantation is the only potentially curative treatment for end-stage lung failure and successfully improves both long-term survival and quality of life. However, lung transplantation is limited by the shortage of suitable donor lungs. This discrepancy in organ supply and demand has prompted researchers to seek alternative therapies for end-stage lung failure. Tissue engineering (bioengineering) organs has become an attractive and promising avenue of research, allowing for the customized production of organs on demand, with potentially perfect biocompatibility. While breakthroughs in tissue engineering have shown feasibility in practice, they have also uncovered challenges in solid organ applications due to the need not only for structural support, but also vascular membrane integrity and gas exchange. This requires a complex engineered interaction of multiple cell types in precise anatomical locations. In this article, we discuss the process of creating bioengineered lungs and the challenges inherent therein. We summarize the relevant literature for selecting appropriate lung scaffolds, creating decellularization protocols, and using bioreactors. The development of completely artificial lung substitutes will also be reviewed. Lastly, we describe the state of current research, as well as future studies required for bioengineered lungs to become a realistic therapeutic modality for end-stage lung disease. Applications of bioengineering may allow for earlier intervention in end-stage lung disease and have the potential to not only halt organ failure, but also significantly reverse disease progression.

## 1. Introduction

**Figure 1:**
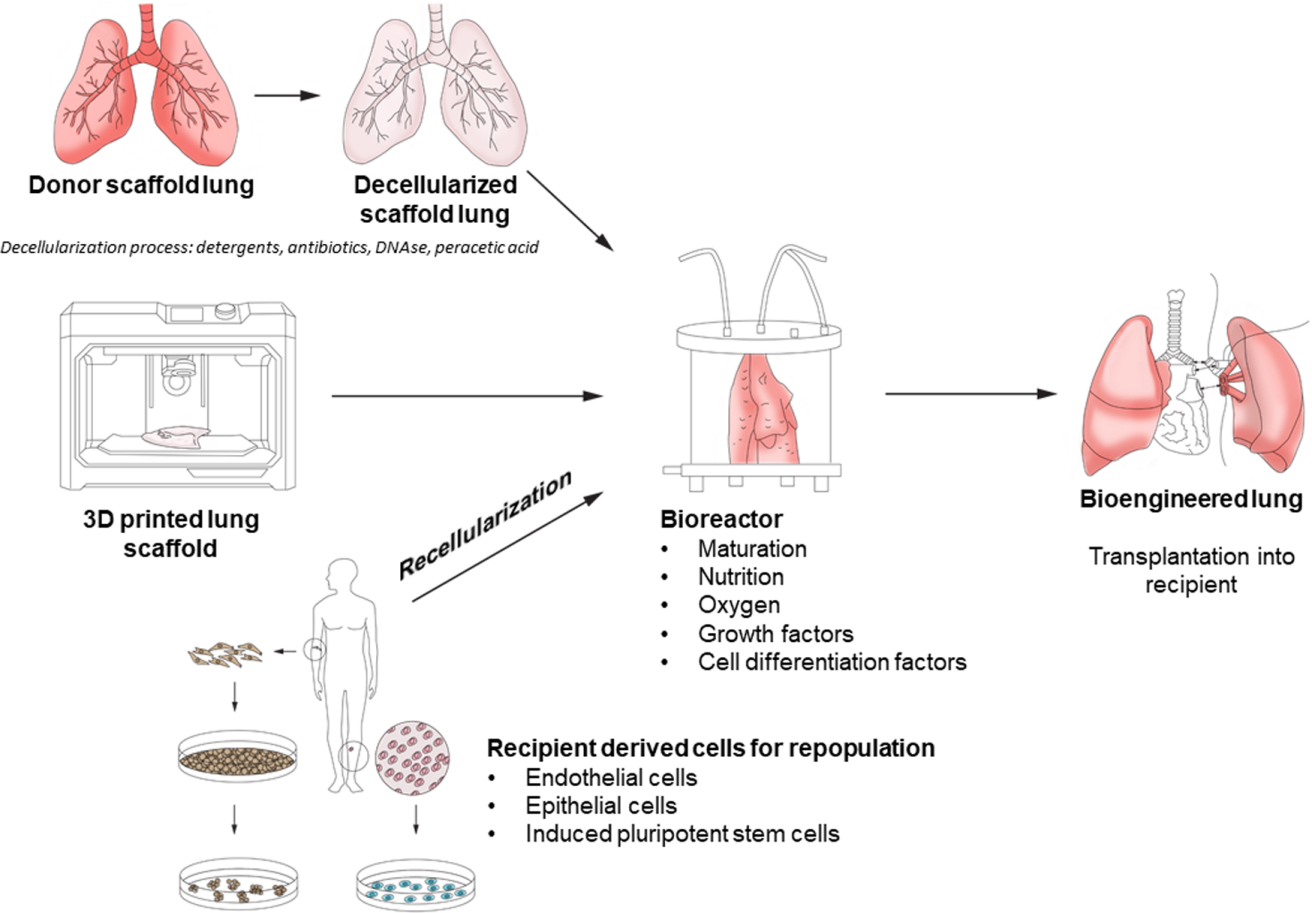
Overview of lung bioengineering process.

Within the field of end-stage lung disease (ESLD), the conventional treatment of choice is lung transplantation. This therapy is limited by an imbalance between the availability of suitable donor lungs and demand, creating a lengthy waiting list. Therefore, alternatives to conventional transplantation to alleviate donor organ shortage have been investigated, such as use of organs from different species (xenotransplantation) as well as bioengineering or regenerative medicine. The basic concept of bioengineering requires the creation of a form of tissue scaffold or matrix, populated by cells of the desired tissue, to transplant into the patient and allow for function as closely to the native organ as possible (Figure 1) (1). If successful, the promise of this approach lies in the ability to replace diseased and damaged lungs with bio-compatible tissue on demand. Tissue engineering approaches have had successful applications in human skin substitutes, vascular grafts, and bladder tissue (2–4). However, lung tissue engineering is made more challenging given the multiple cell types involved (e.g., over 40 different cell types in the lung), the need for structural and vascular membrane integrity, and in the case of lung tissue, ability to perform adequate gas exchange and the mechanical forces of respiration (5).

## 2. Selection of the tissue scaffold

The ideal scaffolding for the bioengineered lung has not yet been established. In tissue engineering, the extra-cellular matrix (ECM) is critical for maintaining mechanical support of the regenerating tissue, establishing the optimal microenvironment for tissue repair and regeneration, and incorporating biochemical cues for the modulation of cell behavior (6). Possible sources for such scaffolding include human tissue, animal tissue, or synthetic materials. Use of human or animal tissue requires a process of decellularization to remove antigenic material which may cause an immune reaction in the recipient.

One possible source of human tissue that may be used for scaffolding are donation after circulatory death (DCD) donors whose lungs are unsuitable for allotransplantation (7–11). A significant problem with the standard transplantation of DCD donor lungs is the length of ischemic time. At 18 h of cold ischemic time (CIT), ischemia-reperfusion injury (IRI) can significantly alter phenotypic features of lung cells—epithelial cells more so than endothelial cells (12). It is plausible that DCD lungs deemed to be untransplantable with prolonged ischemic time can be used as scaffolds for bioengineered lungs. There have also been studies on the use of human scaffolds from older donors with pre-existing lung diseases such as chronic obstructive pulmonary disease (COPD). However, these scaffold sources have demonstrated difficulty sustaining prolonged viability after recellularization compared to human lungs without COPD, suggesting that these cohorts of human lungs may not be an ideal choice (8, 13).

The ECM that remains after lung decellularization provides both structure and biophysical cues for whole organ regeneration after recellularization. A study by Gilpin et al*.* evaluated epithelial stem cells isolated from adult human lung tissue that were cultured on acellular ECM derived from neonatal or adult lung donors (14). There were significantly higher cell proliferation and survival rates for stem cells cultured on neonatal lungs. This may be because the ECM in the neonatal lung is actively undergoing alveologenesis and possesses distinct signals that aid in cell proliferation. The same study also found that treatment of the scaffolds with Fibrillin 2 (FBN-2) and Tenascin C (TN-C) prior to re-epithelization increased epithelial proliferation and tissue remodeling. This finding suggests that FBN-2 and TN-C (identified in neonatal ECM) have an important role in sustaining and maintaining cellular proliferation which is critical for tissue engineering of lung tissue.

Scaffolds need not be of human origin, and decellularized scaffolds from both porcine and non-human primates have been described (15, 16). Primate tissue is the most similar to human in matrix composition, but its use is associated with concerns related to animal welfare (17). Porcine lungs have the advantage of being readily available, and their use is potentially more acceptable in the eyes of the public compared to non-human primates. However, porcine models are susceptible to the formation of blebs and cystic spaces following the decellularization process (9). Furthermore, the composition of the ECM in porcine scaffolds has been shown to be significantly different, possibly inhibiting the proliferation of human endothelial cells (17). Although there is much care taken to decellularize scaffolds to avoid rejection from the recipient, there still exist risks of rejection from other aspects of the ECM. Proteoglycans and collagen in xenogenic ECMs possess antigenic properties and stimulate the production of anti-non-gal antibodies due to differences in protein sequences between human and non-primate ECMs (18, 19). A study by Tao et al*.* demonstrated evasion of the immune system by coating autologous red blood cell membrane on xenogeneic ECM-based tissue engineering graft surface (19). This may be a promising avenue for further research.

Synthetic scaffolds made of poly lactic-co-glycolic acid (PLGA), poly-L-lactic-acid (PLLA), and poly-DL-lactic acid have been studied as well, although all have shown difficulty in guiding correct cell differentiation (20, 21). The use of Matrigel and laminin are promising in addressing the problems in differentiation in murine embryonic stem cells, but have yet to be applied to human embryonic stem cells (22). Due to the fewer number of cell types present in tracheal tissue, more research has been done into producing synthetic tracheal scaffolds using 3D printable materials such as polyhedral oligomeric silsesquioxane poly (carbonate-urea) urethane (POSS-PCU) and polyglycolic acid, pluronic F-127 (23, 24). While these inks are accurate in printing, they lack integrin binding sites and an environment for the biochemical processes required for lung tissue (11). Interestingly, in an effort to more closely mimic alveolar structure for the purpose of lung cancer drug development, materials such as gelatin mixed with microbial transglutaminase have been preliminarily studied and shown to evenly distribute cells and are able to be efficiently seeded (25).

### 2.1. Decellularization protocols and decellularized scaffolds

The aim of the decellularization process is to preserve the airway and vascular structures whilst removing all cells and cellular material yet still preserving as much native ECM as possible. Most protocols for decellularization utilize a detergent solution such as Triton X-100 and sodium deoxycholate or 3-[(3-cholamidopropyl)dimethylammonio]-1-propanesulfonate (CHAPS) detergent perfused through the pulmonary artery and trachea, followed by DNAse and peracetic acid to disinfect and dissociate DNA from the extracellular matrix (26–28). The current criteria of complete decellularization from Gilpen et al*.* includes (i) <50 ng double-stranded DNA per mg of decellularized material; (ii) less than 200 bp length of DNA fragments; (iii) preservation of structural proteins of ECM; and (iv) retention of mechanical properties (29). Confirmation of decellularization can be obtained through DNA quantification techniques (fluorescent nucleic acid stains) and gel electrophoresis to determine DNA fragment size (30). Trace amounts of DNA remain after processing, however, the inflammatory or other responses to small fragments of remnant DNA (<300 base pairs) are unlikely to have clinical effects (31). There is a delicate balance between the goals of removing cellular material and disrupting the mechanical integrity of the ECM with the decellularization process.

Currently, human scaffolds and natural cell-derived ECM matrices are superior to synthetic biomaterials derived hydrogels. However, degradation rates of biomaterials scaffolds are difficult to control, and it is important to choose the right method of decellularization in order to preserve as much of the native ECM as possible. One method of decellularization is through the application of direct pressure to the tissue. Mechanical force is not ideal for many types of tissues which exhibit densely organized ECMs. However, in organs like the lung, mechanical force may be a feasible way to decellularize tissue while maximally preserving the ECM (30). Mechanical methods of decellularization avoid the toxicity of chemical methods and allow surfactant to remain in the decellularized tissue (29).

One of the problems that has been seen during decellularization in whole organs is the loss of the endothelium, resulting in an activation of the coagulation cascade. Consequently, direct contact between the recipient's blood and the ECM of the blood vessels occurs and subsequent intravascular blood coagulation can manifest after implantation and significantly reduces short-term graft survival (32–35). To address this, studies have utilized re-endothelialization of the graft, heparin immobilization on the ECM, end-point immobilization, layer-by-layer techniques, and use of heparin-gelatin to coat the vascular network—all of which have promising results but lack long-term graft survival data (33–37). A recent study from Akinnnola et al*.* explores endothelial cell specifications necessary to prevent thrombus formation and barrier leaks. The group successfully identified one cell source in a mouse study that was able to accomplish the aforementioned characteristics: pulmonary microvascular endothelial progenitor cells. Fathi et al. has also utilized pre-vascularization methods by axial vascularization and subsequent infusion of either bone marrow or adipose-derived stem cells through the portal vein of the Lewis rat liver scaffold after initial decellularization and implantation. The bone marrow and adipose-derived stem cell groups were functionally superior to the acellular scaffold (38).

One of the ways to overcome the challenge of repopulating the lung with so many cell types is to de-epithelialize instead of fully decellularize the lung. In diseases that primarily affect the conducting airways, de-epithelialization may allow for autologous lung transplantation after specific removal of diseased cell populations (39). Dorrello et al*.* describes a method of de-epithelization through infusing a CHAPS detergent solution through the airways while using low tidal volume ventilation to optimize distribution of the solution through the airways which was tested and successful in rat lungs (40). Subsequent targeted reseeding of the epithelium using normal epithelial cells or mutation-corrected iPSCs has the potential to allow patients to keep their native lungs which would result in a lower burden on the lung transplant list (41). This is especially viable now that the technology regarding ex-vivo lung perfusion (EVLP) and cross-circulation (XC) has advanced to a level where the possible amount of time on EVLP or XC supersedes the time needed to de-epithelialize and reseed the lung as proven in large animal models on EVLP and both small and large animal models on XC (40, 42–44). Dorrello et al*.*'s approach to reseeding the epithelium can also be applied to lungs that do not qualify for traditional or extended criteria donation, and would otherwise serve as scaffolds for lung bioengineering. With majority of donor lungs unable to meet the standards for transplantation due to injury located in the alveolar epithelium, de-epithelialization of the donor lung and subsequent reseeding of the lung with recipient stem cells would allow for an increased number of donor lungs to successfully be used as scaffolds. By selectively decellularzing the epithelium, there is the potential to address the problems of recellularization of a wide range of cell types, preservation of the vascular network, and support and delivery of growth factors and signaling molecules vital to the success of lung scaffolds (39).

### 2.2. 3D Printed scaffolds

Recent advances in lung tissue engineering involve production of a synthetic acellular matrix. 3D “bioprinting” is a technology that uses computer-aided models and specific 3D bioprinters with either biomaterials (non-cellular biological material hydrogels such as collagen and gelatin which need to be seeded after printing) or bioinks (which contain biologically active cells that can be processed and printed to produce a biological model) (45). There are challenges with using either. Bioinks must be utilized within temperature and pressure parameters that allow the cells to survive the printing process (45). Scaffolds made with biomaterial inks must be able to sustain cellularization, with the ability to seed on varied cell types. Due to limitations of 3D printers and differences between capabilities in viscosity of materials that can be printed, clogging of the nozzle has often been problematic and limits the number of cell types available to be printed (46, 47).

One promising biomaterial is human recombinant type 1 collagen, which has been produced by crossbreeding tobacco plants to incorporate human genes encoding for procollagen type I, with similar biofunctionality compared to human tissue-derived collagen (48). 3D bioprinting technology has been able to produce models of the alveolus, including endothelial cell, basement membrane, and epithelial cell layers (49). Selection of the most appropriate biomaterial for a synthetic scaffold is challenging. Completely synthetic materials can be altered for varying mechanical properties, an important consideration when supporting respiration, but offer relatively poor cell adhesion and lack biological signals for differentiation and proliferation. Naturally derived hydrogels have the benefit of biocompatibility and biochemical cues for adhesion, although they tend to have poor mechanical properties. Consequently, a mixture of both synthetic materials and naturally derived hydrogels may retain the benefits of each (11, 45).

At the alveolar level, Grigoryan and colleagues developed a model of stereolithographic production of a hydrogel that contained intricate and functional vascular architectures, which subsequently modeled into alveolar structures that could support ventilation cycles for over 6 h (50). This advance proved the ability to produce highly intricate biologically active structures, a previous limitation of 3D printing technologies.

## 3. Bioreactors

Repopulating the scaffold with a suitable lung cell population is another challenge. Ideally, autologous cells from the transplant recipient would be used to recellularize the scaffold. The advantage of this would be complete biocompatibility without the need for immunosuppressive medications. However, adult cells are difficult to obtain from patients in sufficient numbers. Induced pluripotent stem cells (iPSCs) are a promising cell lineage that offer some benefits in bioengineered lungs. iPSCs are obtained from skin fibroblasts, and differentiation of iPSCs into human fetal lung tissue and population of a human scaffold of alveolar epithelial cells have been reported (51, 52). Development of endothelial cells (also necessary for complete recellularization) through reprogramming has been reported in decellularized vascular scaffolds (53). What remains unknown is whether all cell types necessary for recellularization are present in current models. Interstitial cells make up 30% of the lung (such as fibroblasts) and are vital for supporting epithelial and endothelial cell populations. Mesenchymal stem cells, derived from bone marrow, play a role in lung injury and fibroblasts assist in ECM turnover. Excessive fibroblast activity contributes to the development of pulmonary fibrosis (54). Consequently, it is likely that repopulation with interstitial cells (from mesenchymal stem cells and fibroblasts) are necessary for successful lung tissue engineering (55).

Repopulating either synthetic or decellularized scaffold requires a bioreactor. This allows seeding of the cells, adequate nutrient transfer, and waste removal, and additionally provides the appropriate mechanical and biochemical stimuli for cell differentiation. Current technology for human EVLP utilized in clinical transplantation allows for lung perfusion up to 12 h experimentally and 4 h clinically (56, 57). It is likely that repopulation and maturation of a decellularized scaffold will require more time than this, and therefore further research into the development of appropriate bioreactors for human bioengineered lung is necessary.

One of the adverse events of decellularizing organs is the damage to critical ECM components such as elastic fibers, proteoglycans, and glycosaminoglycans. These elements are crucial for tissues to sustain compressive forces, and thus must be a consideration when evaluating bioreactor systems. While some bioreactor systems can recover some of the lost glycosaminoglycans, there is growing evidence that the recipient's body may serve as a superior bioreactor to restore some of the constructs lost during decellularization. This is especially applicable in partial lung transplants, such as single-lobe or tracheal transplants. Tan et al*.* implanted a tissue-engineered bronchus in a lung cancer patient, and at four month follow-up confirmed complete revascularization and re-epithelialization of the bronchus (58). While the patient's death was reported 13 months postoperatively, this nevertheless demonstrated the success of using the body as a bioreactor.

It is important to mimic *in vivo* conditions (mechanical stress, environment) as closely as possible when reseeding the scaffold. This permits the scaffold to maintain the correct signaling and differentiation of cell types in the lung. Due to the variety of cell types that exist in the lung, it may be plausible that synthetic scaffolds be constructed of mixed materials or inks that are optimal in supporting the respective cell types in that area of the lung (59–62).

## 4. Animal studies

There have been numerous animal transplants performed with bioengineered lungs (Table 1). In large animals comparable to humans, the longest survival report to date is from Nichols and colleagues who performed transplantation of porcine lungs using a decellularized pig scaffold (69). They reported survival of one animal to 2 months. While only the airway was anastomosed in this experiment, vascularization of the airway was observed. Yanagiya et al*.* reported 3 pigs who received bioengineered lungs, although all developed issues with gas exchange and bullous emphysema (70). This was potentially due to the degradation of elastin during the decelluarization process. A report from Zhou et al*.* noted reduced lung compliance and gas exchange in the bioengineered lungs, possibly as a result of both the lack of pulmonary surfactant production and immature barrier function (71). Kitano et al*.* observed thrombus formation in the pulmonary artery and vein in pigs at 24 h, with collapse despite high airway inspiratory pressures of 30 cm H_2_O in two pig recipients of bioengineered lungs (72). This was likely due to cross-species hyperacute dysfunction and activation of coagulation cascade despite three drug immunosuppression.

**Table 1:** Summary of bioengineered animal transplantation experiments and survival.

Author	Animal	*n*	Bioreactor culture time	Recellularization cell line	Survival	Anastamosis	Comments on graft function
Ott (63)	Rat	3	9 days	Human umbilical cord endothelial cells and human alveolar basal epithelial cells	6 h	Airway and vascular anastamosis	Development of pulmonary secretions, possibly associated with lack of lymphatic drainage, capillary leak of immature vessels, and ventilation trauma
Doi (64)	Rat	3	8 days	eGFP-labeled vascular endothelial cells, QDs655-labeled adipose-derived stromal cells	3 h	Airway and vascular anastomosis	Pulmonary edema and alveolar hemorrhage, likely caused by incomplete establishment of vascular barrier in endothelial cells group; excessive ASC proliferation in EC-ASC-FGF9 group resulting in vessel obstruction
Petersen (28)	Rat	4	8 days	Neonatal rat lung epithelial cells and microvascular lung endothelial cells	45 min–2 h	Airway and vascular anastamosis	No acute graft failure
Song (65)	Rat	12	7 days	Human umbilical cord endothelial cells	14 days	Airway and vascular anastamosis	One death at 4 h due to pulmonary edema secondary to fungal infection at time of transplantation; defective mucociliary apparatus caused by incomplete proximodistal epithelial cell differentiation
Gilpin (66)	Rat	5	10 days	Human ventralized iPSC derived endothelial cells	60 min	Airway and vascular anastamosis	No acute graft failure
Ren (10)	Rat	6	8 days	Human umbilical vein endothelial cells and human mesenchymal stromal cells (*n* = 3) Human induced pluripotent stem cells (*n* = 3)	3 days	Airway and vascular anastamosis	Low delivery efficiency from fluid driven hydrostatic pressure loss
Obata (67)	Rat	16	3 days	Rat adipose derived stromal cells and rat lung microvascular endothelial cells	30 min	Vascular anastomosis only	Regarding decellularization, potassium laurate (a natural detergent) reduces lung ECM damage when compared to traditional sodium dodecyl sulfate
Jensen (68)	Mouse	Unknown	24 or 50 h	Predifferentiated murine embryonic stem cells	14 days	Implanted subcutaneously	Rapid detergent-based decellularization maintains architecture and critical ECM components and enables maintenance of murine embryonic stem cell differentiation both *in vitro* and after subcutaneous implantation
Nichols (69)	Pig	6	30 days	Adult pig derived lung cells from pneumonectomy	2 months	Airway anastomosis only	Pig 2 euthanized due to respiratory complications at 10 h, Pig 5 suffered from airway occlusion after bioengineered lung transplant
Yanagiya (70)	Pig	3	3 weeks	Autologous airway epithelial cells and vascular endothelial cells obtained by wedge resection 3 weeks prior to transplantation	2 h	Airway and vascular anastomosis	Bullous changes and poor/insufficient carbon dioxide gas exchange
Zhou (71)	Pig	3	6 days	Human umbilical vein endothelial cells and human airway epithelial progenitor cells	1 h	Airway and vascular anastomosis	Stiffer lungs due to lack of pulmonary surfactant, lower level of gas exchange, and immature barrier function relative to native lungs
Kitano (72)	Pig	3	6 days	Human umbilical vein endothelial cells and basal endothelial stem cells	24 h	Airway and vascular anastomosis	Thrombosis and subsequent occlusion of pulmonary arteries

Animal transplantation experiments with bioengineered 3D-printed artificial lung scaffolds are ongoing, however, peer reviewed data has yet to be published (73). While these experiments are in the early developmental stages, there is incremental progress in the use of bioengineered lungs utilizing decellularized scaffolds or artificially manufactured scaffolds. Further work is needed to determine the optimum protocol for de- and re-cellularization and bioreactor maturation. If successful, this regenerative technique promises not only to provide a personalized lung organ replacement, but can also likely be applicable to other organs and can shift the transplantation paradigm away from allo- and xenotransplantation.

## 5. Clinical and human studies

While whole bioengineered lung studies have yet to be done, there have been advances in tracheal and vascular human studies (74, 75). The first successful tissue-engineered vascular graft was a pulmonary artery in 1999 (76). The scaffold was an autologous peripheral vein and showed ability to grow with the patient. In 2008, Macchiarini et al*.* performed a tracheal transplant using a tracheal scaffold of a human donor that had been first decellularized and then recellularized with the recipient's autologous bronchial epithelial cells from bronchoscopic biopsy. A novel bioreactor was used to mimic the biomechanical cues of shear stress. The recipient did not suffer any complications and had normal lung function tests at two-month follow-up. At one month, “the appearance of the graft was indistinguishable from native trachea, and local mucosal bleeding was elicited when the biopsy sample was taken, indicating successful revascularization” (77). However, subsequent events surrounding this transplant and other tracheal transplants have cast significant doubt into the scientific integrity and success of these experimental procedures.

As mentioned previously, in 2015, Tan et al*.* addressed the challenge of the extensive (multiweek) revascularization timeframe associated with implanted tissue engineering (58). During the traditional revascularization timeframe, majority of the cells seeded onto the scaffold die, decreasing viability of the implanted scaffold (78). By using an “in-vivo bioreactor” which is bioengineered tissue—or in this case, porcine acellularized dermix matrix—perfused with continuous medium, the group allowed the pre-seeded cells to remain alive during revascularization while simultaneously reseeding the system and allowing for the addition of various growth factors into the perfusate. During the procedure, the left upper lobe, lower superior segment, and left main bronchus were resected and the engineered left bronchus substitute implanted with two PORT-A-CATH (Smiths Medical, London, UK) inserted in between the two matrix layers. Ringer's solution containing gentamicin was continuously pumped into the scaffold for a month while total nucleated cells were injected directly into the matrix twice a week for a month. The group confirmed revascularization and re-epithelialization 4 months postoperatively which showed the efficacy of the patient serving as his own bioreactor for the scaffold. The patient who initially was given a prognosis of 3 months died at 13 months post-op due to a lung cancer relapse.

## 6. Towards the development of a bioartificial lung

There has been much research regarding prolonging the direction of mechanical circulation and gas exchange in the context of lung injury and bridge to transplant. Along with the increasing use of extracorporeal membrane oxygenation (ECMO) as a bridge to lung transplant, it is possible to envision a device that goes beyond a bridge and serves as the lung itself. In lung transplant patients, longer term use of ECMO as a bridge to transplantation has been associated with worse post-transplant survival rates (79, 80). There are also many complications associated with long term use of ECMO that would also be concerns for an ECMO-type bioartificial lung, such as vascular complications, organ dysfunction (such as kidney injury) due to alteration in blood flow characteristics, oxygenator degradation and malfunction, major bleeding, and significant infection (81). The requirement for battery power (for pump systems) and a continuous oxygen supply are also limitations. Now more than ever, it is crucial to find an accessible and suitable alternative to orthotopic lung transplantation to fulfill the gap between available organs and patients on the waitlist. In 2020, the lung transplant waitlist mortality was 16.1 deaths per 100 waitlist years in the United States, with even more occurring in other solid organ transplantation waitlists (82). Over 2,600 new candidates were added to the lung transplant waiting list that year. Waitlist mortality in other countries with differing policies and availability of donor organs is even higher (83, 84). The field of bioengineering offers unparalleled potential to drastically extend life expectancy in patients with ESLD who may otherwise have no options for salvage therapy.
